# Resistance of Permafrost and Modern* Acinetobacter lwoffii* Strains to Heavy Metals and Arsenic Revealed by Genome Analysis

**DOI:** 10.1155/2016/3970831

**Published:** 2016-10-04

**Authors:** Sofia Mindlin, Anatolii Petrenko, Anton Kurakov, Alexey Beletsky, Andrey Mardanov, Mayya Petrova

**Affiliations:** ^1^Institute of Molecular Genetics, Russian Academy of Sciences, Kurchatov Sq. 2, Moscow 123182, Russia; ^2^Institute of Bioengineering, Research Center of Biotechnology of the Russian Academy of Sciences, Leninsky Ave. 33, Bld. 2, Moscow 119071, Russia

## Abstract

We performed whole-genome sequencing of five permafrost strains of* Acinetobacter lwoffii* (frozen for 15–3000 thousand years) and analyzed their resistance genes found in plasmids and chromosomes. Four strains contained multiple plasmids (8–12), which varied significantly in size (from 4,135 to 287,630 bp) and genetic structure; the fifth strain contained only two plasmids. All large plasmids and some medium-size and small plasmids contained genes encoding resistance to various heavy metals, including mercury, cobalt, zinc, cadmium, copper, chromium, and arsenic compounds. Most resistance genes found in the ancient strains of* A*.* lwoffii* had their closely related counterparts in modern clinical* A*.* lwoffii* strains that were also located on plasmids. The vast majority of the chromosomal resistance determinants did not possess complete sets of the resistance genes or contained truncated genes. Comparative analysis of various* A*.* lwoffii* and of* A*.* baumannii* strains discovered a number of differences between them: (i) chromosome sizes in* A*.* baumannii* exceeded those in* A*.* lwoffii* by about 20%; (ii) on the contrary, the number of plasmids in* A*.* lwoffii* and their total size were much higher than those in* A*.* baumannii*; (iii) heavy metal resistance genes in the environmental* A*.* lwoffii* strains surpassed those in* A*.* baumannii* strains in the number and diversity and were predominantly located on plasmids. Possible reasons for these differences are discussed.

## 1. Introduction

The presence of plasmids, extrachromosomal self-replicating circular DNA molecules, in most bacterial species is connected to their key role in rapid adaptation to changing environmental conditions without altering the gene content of the bacterial chromosome. Typically, in addition to few essential genes a plasmid harbors genes coding for ecologically important properties such as variable metabolic processes, nitrogen fixation, resistance to antibiotics and heavy metals, and pathogen virulence.

In recent years, with the revolution in sequencing technologies the number of sequenced bacterial plasmids increased dramatically and reached about 4600 [1], but only a small number of these plasmids have been analyzed in detail. In particular, not enough attention was paid to such important issues as evolution and dynamics of plasmids in natural bacterial populations, despite a number of works that were devoted to this research [2–7]. Bacteria belonging to the* Acinetobacter* genus are a convenient model for such studies since these are ubiquitous bacteria that play an important role in different ecological niches including soil, water, and association with animals and, at the same time, some of them are common human pathogens. For instance, Fondi et al. [8] have undertaken a study of evolutionary relationships between 29* Acinetobacter* plasmids using the information on sequenced plasmids. It should be noted that the authors included in the list of analyzed sequences eight plasmids containing deficient mercury resistance transposons described in our previous work [9]. The authors demonstrated the important role of rearrangements between different plasmids and concluded that transposases and selective pressure for mercury resistance have played a pivotal role in plasmid evolution in* Acinetobacter*. Unfortunately, only small regions (less than 10%) of the mercury resistance* Acinetobacter* plasmids were sequenced at that time and this fact was not taken into account. In addition, the selected plasmids did not represent adequately the diversity of plasmids hosted by different strains belonging to the* Acinetobacter* genus.

Recently, we could continue our studies on the molecular structure and phylogenetic relations of* Acinetobacter*'s plasmids using next-generation sequencing methods. For our studies we have chosen five ancient strains belonging to* Acinetobacter lwoffii*, guided by the following considerations: (i) in comparison with plasmids of* A*.* baumannii,* plasmids of* A*.* lwoffii* have been studied very little; (ii) in the last years,* A*.* lwoffii* strains were included in the list of dangerous pathogens to humans.

Surprisingly, analysis of genomic sequences demonstrated that all the ancient strains isolated from uncontaminated permafrost sediments contained plasmids with genes of resistance to salts of heavy metals and arsenic. We hypothesized that not only mercury but also other heavy metals have played and continue to play an important role in the evolution of* Acinetobacter*. This prompted us to study the occurrence and abundance of such genes in the genomes of* A*.* lwoffii* isolates. Here we describe the results of analysis of the structure and distribution of heavy metals and arsenic resistance genes found in the genomes of ancient as well as of modern strains of* A*.* lwoffii*.

## 2. Materials and Methods

### 2.1. Bacterial Strains and Growth Conditions

The ancient* Acinetobacter lwoffii* strains used in this study (Table 1) were previously isolated from permafrost sediments collected from different regions of Kolyma Lowland [9, 10]. All strains were grown in lysogeny broth (LB) medium or solidified LB medium (LA) [11] at 30°C.

### 2.2. Whole-Genome Sequencing and Assembly of Plasmids

The genomes of the ancient* A*.* lwoffii* strains were sequenced with a Roche GS FLX genome sequencer (Roche, Switzerland) using the Titanium protocol to prepare shotgun genomic libraries. Approximately 40-fold sequence coverage was achieved for each genome. The GS FLX reads were assembled into contigs using the GS De Novo Assembler (Roche); among them, we identified those contigs that contained genes for mobilization and/or replication of plasmids. PCR was used to close the gaps between assembled plasmid contigs and to confirm their circular structure.

### 2.3. Bioinformatics Analysis

For the assembly and analysis of plasmid genomes from ancient strains the program UGENE (http://unipro.ru/) was used. Similarity searches were performed using BLAST [12] and REBASE [13]. Open reading frames (ORFs) were searched using ORF Finder and BLAST software at NCBI. Conserved domains and motifs were identified using the NCBI Conserved Domain Database (CDD) [14] and the Pfam database [15]. Contigs from the whole-genome shotgun sequences of clinical* A*.* lwoffii* strains were considered chromosomal if their size exceeded 300 kb and if they contained housekeeping genes. Relatively small (less than 200 kb) contigs were attributed to plasmids or their parts if they contained determinants typical for plasmids and/or if they contained no housekeeping genes.

### 2.4. Standard DNA Manipulations

Standard protocols were used for DNA isolation and agarose gel electrophoresis [11]. PCR was performed with a Mastercycler (Eppendorf) using Taq DNA polymerase and Long PCR Enzyme Mix (for amplification fragments more than 6 kbp) with supplied buffers (Thermoscientific), dNTP mixture (Thermoscientific), and appropriate primers. Amplified DNA fragments were sequenced using conventional Sanger method (Applied Biosystems 3730 Genetic Analyzer) at the Interinstitute Genome Center (Moscow).

### 2.5. Determination of the MICs for Heavy Metals and Arsenic Salts' Resistance

The level of resistance was determined by the agar dilution method [16]. The MICs of heavy metals and arsenic salts for the ancient* A*.* lwoffii* strains were determined. Bacterial strains were grown in LB broth at 30°C with shaking for 3 h and diluted 100-fold with fresh broth. 5 *μ*L of the bacterial suspension (about 5 × 10^6^ cells per mL) was plated with a bacteriological loop onto LA (to determine the MICs of As3, As5, Hg, and Cr) or Adams [11] (to determine the MICs of Cu, Co, Cd, and Zn) plates supplemented with heavy metals and arsenic salts. The used salts and their concentrations (mM) tested were as follows: AsNaO_2_ (As3): 5, 10, 20, and 30; Na_2_HAsO_4_  ×  7H_2_O (As5): 20, 40, and 60; K_2_Cr_2_O_7_ (Cr): 0.3, 0.6, 0.9, 1.2, 1.5, and 1.8; CuSO_4_  ×  5H_2_O (Cu): 0.9, 1.8, 2.7, 3.6, and 4.0; HgCl_2_ (Hg): 0.015, 0.03, and 0.06; CdCl_2_  ×  H_2_O (Cd): 0.01, 0.025, and 0.05; CoCl_2_  ×  6H_2_O (Co): 0.01, 0.05, and 0.1; ZnSO_4_  ×  7H_2_O (Zn): 0.2, 0.4, 0.8, 1.6, 3.2, and 6.4; NiSO_4_  ×  7H_2_O (Ni): 0.45, 0.9, 1.8, and 2.7. The plates were incubated at 30°C for 24–30 h and visually inspected.

## 3. Results and Discussion

### 3.1. Whole-Genome Sequencing of Five Ancient Permafrost Strains of* A. lwoffii* and Identification of the Resistance Plasmids

During analysis of the whole-genome shotgun contigs of five ancient permafrost strains of* A*.* lwoffii* (Table 1), we paid the main attention to assembly and identification of plasmids. In total, to date we have identified 35 plasmids. It was found that 4 of 5 bacterial strains contained 8–12 plasmids and one strain carried 2 plasmids. The plasmids varied considerably in size (from 4,135 to 287,630 bp) and structural features. In our previous work, we identified and described in detail one of the small plasmids, pALWED1.8 with streptomycin/spectinomycin gene* aadA27* [17]. In the present work, we focused on the study of resistance genes to heavy metals and arsenic salts found in all the plasmids. Therefore, we first identified the plasmids harboring genes encoding resistance to salts of mercury (*mer* operons), arsenic (*ars* operons), chromium, copper (*cop* locus), and cobalt/zinc/cadmium (*czc* determinants).

Although all strains were isolated from pristine permafrost sediments, each of them contained one or two plasmids carrying different combinations of resistance genes to heavy metals and arsenic salts (Table 2).

We hypothesize that the abundance and diversity of the resistance genes may be associated with the environmental habitat of the ancient strains. We decided to test the functional activity of the detected resistance genes, to analyze the structure of the resistance determinants found in ancient plasmids, and to check the presence of the resistance genes and their localization in genomes of modern strains of* A*.* lwoffii*.

### 3.2. Functional Activity of Resistance Genes

To test the activity of resistance determinants identified in the ancient plasmids the levels of resistance to salts of mercury, arsenic, chromium, copper, cobalt, zinc, cadmium, and nickel of five permafrost* A*.* lwoffii* strains were examined. For this purpose, MICs for different salts were determined by the agar dilution method (see Section 2). We carried out five independent experiments. All strains containing resistance genes were able to grow at higher concentrations of corresponding toxic compounds compared with strains not containing resistance determinants (Table 2). Thus, it can be concluded that at the high probability all detected resistance genes are functionally active. However,* czc* determinants in some strains provided resistance to salts of not all three metals (cobalt/zink/cadmium), but only two of them. For example, the strain ED9-5а was sensitive to cobalt and ЕК30А to zinc salts. These facts can be explained by the existence of a complex transcriptional regulation of different components of the* czc* system described in [18].

### 3.3. Structure of Resistance Determinants Revealed on the Plasmids from Ancient* A. lwoffii* Strains

#### 3.3.1. *Mer* Operons

**Figure 1:**
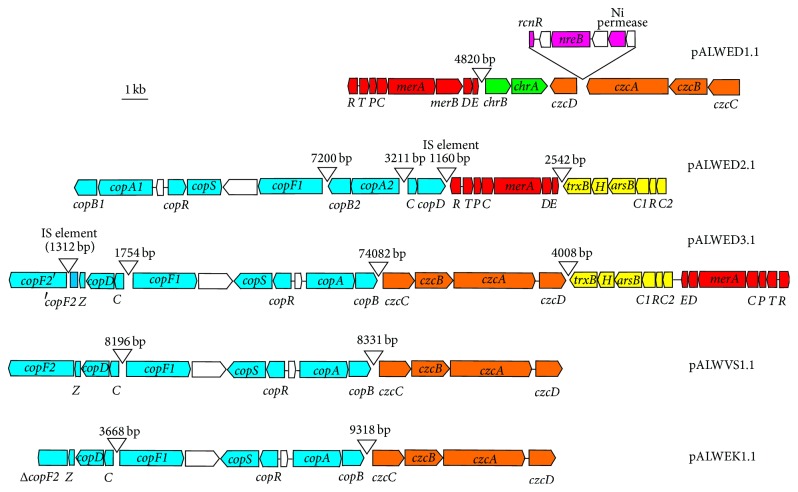
Genetic structure of resistance regions detected in the five large permafrost multiresistance plasmids (scheme). The location and polarity of genes are shown with arrows; genes belonging to* mer* operon are marked in red;* ars* operon in yellow; chromium resistance genes in green;* cop* genes in blue;* czc* in orange; nickel resistance genes in magenta; and genes with unknown functions and foreign genes in white. Numbers show the size of the sequences located between different resistance determinants and genes.

The* mer* operons of Gram-negative bacteria contain a metalloregulator gene (*merR*) and three structural genes: genes that encode a transport system that delivers the toxic mercuric ions into the cells (*merTP*) and a gene that encodes an intracellular enzyme, mercuric reductase (*merA*), which converts mercuric ions into less toxic metallic mercury [19]. Thus, the minimum set of essential mercury resistance genes is* merRTPA*; other genes (*merC*,* merF*,* merD*,* merE*,* orf2*, and* orfY*) occur in various combinations in the* mer* operons of Gram-negative bacteria and are not essential accessory genes. Mercury resistance operons and transposons of* Acinetobacter* strains have been the subject of our previous studies. In particular,* mer* operon of permafrost* A*.* lwoffii* strain ED23-35 studied in this work was described in detail in 2004 [9]. It was shown that in addition to the standard set of* mer* genes (*merRTPCADE*) characteristic to* Acinetobacter* strains it contains gene* merB* encoding organomercurial lyase. In this study, we found* mer* operons in two more strains resistant to mercury, ED45-23 and ED9-5a (Table 3).* Mer* operons of these strains were almost identical to each other and unlike strain ED23-35 did not contain the* merB* gene (Figure 1).

#### 3.3.2. Chromium Resistance Determinants

Genetic analysis of chromate resistant bacteria* Pseudomonas aeruginosa* [20] and* Alcaligenes eutrophus* [21] has shown that main genes encoding the chromium resistance are genes* chrA*, encoding the chromate transporter protein, and* chrB*, encoding chromate resistance protein, probably performing the functions of a regulator. A hydrophobic membrane protein, ChrA, catalyzes the active efflux of chromate (chromate efflux pump) from the cytoplasm and chromate or dichromate induces expression of the* chrA* gene through the action of chrB [22]. It should be noted that, besides* chrA* and* chrB*, some accessory genes have been found in transposons and plasmids of chromate resistance bacteria.

Previously, chromium resistant bacteria were isolated from various groups of the genus* Acinetobacter*, in particular from* A. lwoffii* [23]. However, the resistance determinants in these bacteria were not described.

We revealed the* chrA* and* chrB* genes in three permafrost strains, ED23-35, ED9-5a, and EK30A (Tables 2 and 3). In all these strains chromium resistance genes were located on small plasmids pALWED1.3, pALWED3.5, and pALWEK1.5, respectively, and were almost identical (99% identity at the nucleotide sequence level) on their molecular structure. At the same time, these plasmids differed by their length as well as by the set and structure of essential genes. In particular, plasmid pALWED3.5 contained a* mobA* gene while in two others this gene was absent.

Interestingly, strain ED23-35 contained one more pair of the* chrA*-*chrB* genes. They were located on the largest plasmid pALWED1.1 (Figure 1) and differed significantly from the chromium resistance genes located on the small plasmids in their structure (identity about 60–70% at the nucleotide sequence level). Moreover, we could not reveal genes closely related to this variant of the* chrAB* genes in public databases.

Thus it can be concluded that chromium resistance genes* chrAB* are widely spread in environmental* A*.* lwoffii* strains. Because almost identical genes are present on different plasmids harbored by different* A*.* lwoffii* strains it can be proposed that they can spread via horizontal gene transfer.

#### 3.3.3. Copper Resistance Determinants

Copper is needed for cell metabolism but is toxic at elevated concentrations. This challenge has led to the evolution of complex mechanisms of copper resistance in bacteria. Now a number of different systems that allow bacterial cells to survive in the presence of high concentration of copper have been identified in different taxa of bacteria [24–28].

We found copper resistance determinants on plasmids from four permafrost* A*.* lwoffii* strains. They were located on the large plasmids (pALWED2.1, pALWED3.1, pALWVS1.1, and pALWEK1.1) (Table 3). We studied genetic structure of the resistance determinants harbored by pALWVS1.1 and revealed that they are characterized by unique features. We found that they are closely related to the copper resistance determinants of* Acinetobacter baumannii* AB0057 (CP001182.1) and* Acinetobacter johnsonii* XBB1 (CP010350.1) which are located on their chromosomes and form a single cluster. In addition to genes* copABCD* this cluster contains genes encoding copper-exporting ATPase (*copF*), heavy metal sensor kinase (*copS*), and transcriptional activation protein (*copR*). An unusual characteristic of the copper resistance determinants of* Acinetobacter* strains is the location of the* copAB* and* copCD* genes at a considerable distance from each other, on both sides of other genes involved in copper resistance (Figure 1); in other bacterial species these four genes are linked together and are parts of the same operon [26, 27, 29].

It should be noted that the cluster of copper resistance genes revealed in pALWVS1.1 in comparison with the above-mentioned chromosomal clusters contained an extra fragment about 5000 bp inserted into the region located between the* copC* and* copF* genes (Figure 1). In this fragment we detected genes encoding two transposases and integrase. The same clusters of copper resistance genes were detected in pALWED3.1 and pALWEK1.1.

The genes of copper resistance found in pALWED2.1 are closely related to those described above but form a more complex mosaic structure as a result of the insertion of a large DNA fragment (about 12 kb). This insert contained (i) an extra copy of the* copA-copB* genes (with the identity level about 68–74% to the standard copy); (ii) genes encoding cation transporter and methyltransferase; (iii) genes encoding transposases of different IS elements.

To our knowledge, such complex operons of copper resistance have not yet been studied in detail.

#### 3.3.4. Determinants of Arsenic Resistance

To overcome the toxic effect of arsenic compounds bacteria use different mechanisms based on their active extrusion, extracellular precipitation and chelation, and intracellular transformation [30]. Several genetic systems encoding arsenic resistance in Gram-negative bacteria were revealed and studied to date [30, 31]. We studied in detail the structure of plasmids of two permafrost* A*.* lwoffii* strains, ED45-23 and ED5-9a, resistant to arsenic compounds (Table 2). Indeed, in each of these strains we revealed a single plasmid containing genes involved in arsenic resistance. These were plasmid pALWED2.1 (190039 bp) and plasmid pALWED3.1 (138027 bp). In both plasmids we detected almost identical* ars* operons containing six genes:* trxB*,* arsH*,* arsB*,* arsC1*,* arsR*, and* arsC*2 (Table 3 and Figure 1). Based on the analysis of published data it can be suggested that the genes of* ars* operon encode the following polypeptides: organoarsenic oxidase, thioredoxin reductase, oxyanion translocation protein, arsenate reductase, arsenite-inducible repressor, and second arsenate reductase protein [30, 32, 33].

The structure of* ars* operons involved in arsenic resistance in* A. lwoffii* was most similar to those (*ars H*,* arsB*,* arsC*, and* arsR*) described in* P*.* putida* (NC_002947) and in* Herminiimonas arsenicoxydans* [30]. A significant difference was the presence of two additional genes:* trxB* at the beginning of* ars* operon and the* arsC* at the end of this operon. It should be mentioned that to our knowledge such variants of the* ars* operons have not been described yet.

#### 3.3.5. Determinants of Resistance to Co/Zn/Cd

One of the first studied plasmid-encoded transport systems directed on elimination of toxic cations is the* czc* efflux system [34]. The* czc* resistance operon was originally found and studied in plasmid of* A*.* eutrophus* CH34. It was shown that* czc* operon includes four genes encoding four proteins, CzcA, CzcB, CzcC, and CzcD, from which CzcA is the central protein with efflux capacities [35].

We revealed functionally active* czc* operons in four permafrost* A*.* lwoffii* strains: ED23-35, ED9-5a, EK30A, and VS15 (Table 2). The* czc* operon harbored by pALWVS1.1 included the four genes* czcD*,* czcA*,* czcB,* and* czcC* (Table 3 and Figure 1). Closely related* czc* operon was found in the strain EK30A on the plasmid pALWEK1.1 (identity about 97%). These two operons were for convenience designated* czc1*. Two other* czc* operons differed from* czc1* as well as one from another.

The* czc2* operon revealed on the plasmid pALWED1.1 (strain ED23-35) was only distantly related to* czc1* (about 50% identity at aa sequence level). In addition, between genes* czcD* and* czsA* it contained about 4 kb insertion with six ORFs, including three ones encoding the resistance to nickel (*rcnR*,* nreB,* and gene of Ni permease). Accordingly, strain ED23-35, as opposed to other* A*.* lwoffii* strains, was resistant to nickel salt (Table 3 and Figure 1).

The third* czc* operon,* czc3,* located on plasmid pALWED3.1 (strain ED9-5a) differed significantly from* czs1* as well as from* czc2*. This operon was most closely related (97% identity) to the chromosomal* czc* operon of* Moraxella osloensis* CCUG 350 (CP014234), bacterial strain belonging to another genus of Moraxellaceae family. This observation certainly suggests the involvement of horizontal gene transfer in the evolution of heavy metal resistanсe in bacteria.

It should be noted that the* czc2* operon as well as* chrA-chrB* genes located on plasmid pALWED1.1 differed significantly from respective determinants found on the other plasmids. It can be assumed that these differences are due to different functional characteristics of these plasmids: plasmid pALWED1.1 is conjugative, while the two others are not.

### 3.4. Distribution of Resistance Genes in Chromosomes and Plasmids of* A. lwoffii*

We determined the distribution of the resistance determinants found in plasmids of permafrost strains in plasmids of clinical* A*.* lwoffii* strains and in chromosomes of environmental and clinical strains of* A*.* lwoffii*.

We focused on the analysis of contigs obtained in our work during the whole-genome sequencing of ancient* A*.* lwoffii* strains and of whole-genome shotgun sequences of clinical* A*.* lwoffii* strains obtained from public databases. It should be noted that, unlike* A*.* baumannii* strains, no complete genome sequence of* A*.* lwoffii* was published and all whole-genome shotgun sequences of this species belong to clinical strains.

We have found in the clinical strains the whole set of resistance determinants which we revealed on the plasmids of the permafrost strains (see Table  S1 in Supplementary Material available online at http://dx.doi.org/10.1155/2016/3970831). However, there were significant differences between the determinants found on chromosomes and plasmids. The determinants revealed on the plasmids of clinical strains were closely related to those of the permafrost strains plasmids. The chromosomal determinants of all kinds/types formed distinct lineages and were characterized by different features in comparison with plasmid determinants (Table 4).

In particular, we detected on presumably plasmid contigs of clinical* A*.* lwoffii* strains* mer* operons,* ars* operons,* czc* operons,* cop* operons, and* chrA-chrB* genes almost identical (99-100% identity) to those located in the permafrost plasmids (Table  S1). It is important to note also that in the plasmids of the clinical strains the different resistance determinants were linked to each other (Table  S1). For instance, we have found in similar 14 kb contigs of the strains NIPH512 and NBRC 109760 resistance determinants to mercury, arsenic, and chromium and in the strain NIPH 715 (33 kb contig) resistance determinants to mercury, arsenic, and copper (in Table  S1 these contigs are shown in bold). Therefore the linked location of operons involved in the resistance to different heavy metals can be regarded as a characteristic feature of* A*.* lwoffii* plasmids.

Resistance determinants located on bacterial chromosomes differed from the plasmids by several features: (i) the vast majority of chromosomal operons contained incomplete sets of genes or defective operons (the only exception is the* czc* operon and genes* chrA* and* chrB* from the strain CIP64.10 (APQS00000000)) (Table 4); (ii) the proteins encoded by chromosome and plasmid genes shared less than 85% identical amino acid residues (from 44% to 83%) and differed by their size (Table 4). Note that all these features are common to the environmental as well as to clinical* A*.* lwoffii* strains.

To illustrate this situation it is best to consider the data on the structure of chromosomal* ars* operons and their distribution in the chromosomes of* A*.* lwoffii* (Table  S2). It is seen that, in many cases, chromosomal* ars* operons are incomplete (missing one or two from six genes) (Table  S2). In other cases all genes are present, but apparently the operon is functionally nonactive due to nonsense or frame-shift mutations or deletions in some of its genes. To verify the functional activity of chromosomal* ars* operons we compared the resistance to arsenic of ancient* A*.* lwoffii* strains containing plasmid-encoded* ars* operon (ED45-23 and ED9-5a) and strains containing only chromosomal* ars* operons (ED23-35, VS15, and EK30a). All three strains with only chromosomal* ars* genes were sensitive to arsenic, unlike strains containing plasmid operons (Table 2). Thus, ancient* A*.* lwoffii* strains contain active* ars* operons only in their plasmids. However, it can be suggested that chromosomal* ars* operons played an important role in the evolution of* A*.* lwoffii*, because of their presence in multiple copies on chromosomes in ancient as well as in present-day strains of this species.

It should be noted that other chromosomal operons (*cop*,* czc*, and* chrA-chrB*), as a rule, also did not contain a complete set of genes. The* mer* operon was an exception since we failed to detect its chromosomal copies in any of the studied strains of* A*.* lwoffii*.

### 3.5. Comparative Analysis of Genomes of* A. lwoffii* and* A. baumannii*

Our data indicate that functionally active operons of* A*.* lwoffii* encoding resistance to heavy metal salts are preferably located on plasmids and that many strains of* A*.* lwoffii* are characterized by the presence of numerous plasmids, including plasmids larger than 100 kb. To determine whether these properties are also inherent to clinical* Acinetobacter* strains, especially to strains of* A*.* baumannii*, we compared genomic sequences of* A*.* lwoffii* and* A*.* baumannii*, with particular attention to the size of their chromosomes and the number and structure of their plasmids.

For our comparative analysis, we selected a collection of well-studied strains of* A. baumannii* described in the work of Ou et al. [36].

Based on the phylogenetic data, three of the strains (AYE, AB307-0294, and AB0057) belonged to global clone I (GC I). Two of the three BJAB strains (BJAB07104 and BJAB0868), along with 4 previously reported Asia strains, including MDR-ZJ06 (China), MDR-TJ (China), ABTCD0715 (Taiwan), and AB1656-2 (Korean), were grouped together with ACICU, a strain of global clone II (GC II) group. The strain BJAB0715 probably has a different origin in comparison with other drug-resistant strains. Strains ATCC17978 and AB307-0294 were susceptible to antibiotics and strain SDF was isolated from a human body louse [32, 36].

In addition, we analyzed a number of* A*.* baumannii* strains whose genomes were completely sequenced and studied in recent years. In total, we analyzed the genomes of 31 strains of A. baumannii.

Unlike* A*.* baumannii*,* A*.* lwoffii* strains have only recently begun to be actively studied. To date, information on the whole-genome shotgun sequencing of only 7 clinical isolates of* A*.* lwoffii* was deposited to database, but the authors did not perform their analysis. In our work we complemented the existing data by sequencing of the genomes of five ancient* A*.* lwoffii* strains isolated from permafrost deposits.

Analysis of clinical* A*.* lwoffii* genomes demonstrated that average size of the genomes of the present-day clinical strains is about 3.4 Mbp (Table  S3). According to our data, the genomes of permafrost* A*.* lwoffii* strains have similar sizes.

Comparative analysis of well-studied strains of* A*.* baumannii* demonstrated that they have genome size of about 4.0 Mbp and contain no more than 4 plasmids (Table  S4). Despite the differences in the number and quality of the genomic sequences of the two species, the results clearly indicate that the dimensions of the* A*.* baumannii* chromosomes exceeded those of* A*.* lwoffii* by about 20%. Less reliable conclusion is possible about the differences in the number of plasmids present in the strains of* A*.* baumannii* and* A*.* lwoffii* due to the limited data available. It seems that the plasmids in the* A*.* lwoffii* strains are more numerous than in* A*.* baumannii*.

At present, many plasmids found in* A*.* baumannii* strains are identified and sequenced. We have analyzed the structure of 34 medium-size and large plasmids but detected only one plasmid (pD36-4) containing full* mer* operon and one with fragments of* ars* operon (pAB3). At the same time, we detected full resistance operons in chromosomes of many strains of this species. For instance, we revealed* mer* and* ars* operons in the chromosomes of strains AYE and AB0057 [3]; copper operon in the chromosomes of strains LAC-4, ATCC 17978, and AB0057 [31];* czc* operons in the chromosomes of strains ATCC17978 (CP000521), ACICU (CP000863), AB5075-UW (CP008706), AB0057 (CP001182), and AYE (CU459141).

Thus, unlike* A*.* baumannii* plasmids, plasmids of* A*.* lwoffii* carry a big load of functionally active heavy metal resistance determinants; this corresponds to larger numbers and sizes of plasmids in* A*.* lwoffii* compared to* A*.* baumannii* (Table 2 and Table  S4).

The nature of these differences, whether or not they are associated with lifestyles of two species in distinct ecological niches, is a subject for further studies.

## 4. Conclusions

We for the first time sequenced and analyzed several large plasmids (130–280 kb), which encode genes multiresistance to heavy metals and arsenic compounds, in environmental strains of* A*.* lwoffii*, a potential human pathogen. All five large plasmids of* A*.* lwoffii* contained genes of heavy metal resistance in different combinations. In particular, we revealed genes of resistance to mercury, arsenate, chromium, copper, nickel, and cobalt/zinc/cadmium. The* cop* loci and* ars* operon differed by their structure from those described earlier.* Mer* and* ars* operons were linked to each other. We also obtained preliminary data showing that large plasmids encoding for multiresistance to heavy metals are present in clinical isolates of* A*.* lwoffii*. Thus it seems that in* A*.* lwoffii* determinants of heavy metal resistance are often located on plasmids.

A completely different situation was revealed in studies of metal-resistant* A*.* baumannii* strains. We have analyzed completely sequenced genomes of 31* A*.* baumannii* strains from public databases including their chromosomes as well as plasmids. As a rule, heavy metal resistance genes forming full operons were located on chromosomes and only sometimes were they found on plasmids. In most cases, chromosomal resistance genes were related to the plasmid genes found in* A*.* lwoffii* strains. Furthermore, the average chromosome size of* A*.* baumannii* exceeded that of* A*.* lwoffii* by about 20%, which might indicate the higher role of chromosomes of* A*.* baumannii* in their lifestyle in comparison with* A*.* lwoffii*.

The results of our work suggest the occurrence of two different strategies in two species of the same genus revealed by different structural and functional roles of plasmids and chromosomes. One can speculate that these two strategies may be due to the fact that* A*.* lwoffii* strains, unlike* A*.* baumannii*, mainly inhabit natural environments. Furthermore, our data support hypothesis that not only mercury but also other heavy metals and arsenic have great impact on the evolution of* Acinetobacter* genomes. The accuracy of these conclusions should be confirmed by further studies of plasmids harbored by both environmental and clinical strains of* A*.* lwoffii*.

## Supplementary Material

The Supplementary Material contains 4 tables. The data presented in Table S1 indicate that determinants revealed on the plasmids of modern *A.lwoffii* clinical strains are closely related to those of the ancient *A. lwoffii* plasmids. Table S2 contains the data on the structure and distribution of chromosomal ars operons in *A. lwoffii* strains. Tables S3 and S4 contain the information on comparative genome size of the strains of *A.lwoffii* and *A. baumannii*, respectively.

## Figures and Tables

**Table 1:** Characteristics of permafrost *Acinetobacter lwoffii* strains.

Strain	Age of sediment^*∗*^	Sampling locality
ED23-35	20–40 K	Kolyma Lowland, Bank of river Homus-Yuryiah, Late-Pleistocene-Ice-Complex
ED45-23	20–40 K	Kolyma Lowland, Bank of river Homus-Yuryiah, Late-Pleistocene-Ice-Complex
ЕD9-5а	15–30 K	Kolyma Lowland, Bank of river Homus-Yuryiah, Late-Pleistocene-Ice-Complex
ЕК30А	1,6–1,8 M	Kolyma Lowland, Bank of river Konkovaya, Early Pleistocene
VS15	2-3 M	Kolyma Lowland, Bank of river Grand Chukochia, Late-Pleistocene-Ice-Complex

^*∗*^Age of subsoil permafrost sediments (corresponding to the period of freezing before the present) in years: K = 10^3^ years; M = 10^6^ years.

**Table 2:** Characteristics of ancient plasmids carrying heavy metal resistance determinants and corresponding MICs.

Strain	Plasmid [GenBank accession number]	Size of plasmid (bp)	Resistance determinants	MICs^*∗*^
ED23-35	pALWED1.1 [KX426227]	287630	Hg, Chr, Co/Zn/Cd, Ni	**Hg(>0.06)**, As3(<5), As5(20), **Cr(1.8)**, **Co(0.1)**, **Cd(0.025)**, **Zn(6.4)**, **Ni(2.7)**, Cu(<0.9)
	pALWED1.3 [KX426228]	16071	Chr	

ED45-23	pALWED2.1 [KX426229]	190039	Hg, Ars, Cu	**Hg(0.06)**, **As3(20)**, **As5(40)**, Cr(<0.3), Co(<0.05), Cd(<0.012), Zn(<0.2), Ni(<0.45), **Cu(0.9)**

ЕD9-5а	pALWED3.1 [KX528687]	138028	Hg, Ars, Co/Zn/Cd, Cu	**Hg(>0.06**), **As3(30)**, **As5(60)**, **Cr(1.2)**, Co(<0.05), **Cd(0.025)**,** Zn(3.2)**, Ni(1.8), **Cu(3.6)**
	pALWED3.5 [KX426230]	16568	Chr	

VS15	pALWVS1.1 [KX426232]	134767	Co/Zn/Cd, Cu	Hg(<0.015), As3(<5), As5(<20), Cr(<0.3), **Co(0.05)**, **Cd(0.1)**, **Zn(3.2)**, Ni(0.9), **Cu(3.6)**

ЕК30А	pALWEK1.1 [KX528688]	209983	Co/Zn/Cd, Cu	Hg(<0.015), As3(<5), As5(<20), **Cr**(**1.2**), **Co(0.05)**, **Cd(0.025)**, Zn(<0.2), Ni(0.45), **Cu(3.6)**
	pALWEK1.5 [KX426231]	8227	Chr	

^*∗*^The minimal inhibitory concentrations (mM) of each salt are presented in parentheses; resistance due to the presence of the relevant genes marked in bold.

**Table 3:** Heavy metal resistance operons identified in permafrost strains of *A. lwoffii*.

Operon	Operon genes	Plasmid	Closest relative (AC)	Nucleotide sequence identity (%)
*mer*	*merR*, *T*, *P*, *C*, *A*, *D*, *E*, (*B* ^*∗*^)	pALWED1.1 pALWED2.1 pALWED3.1	*A. lwoffii*, pKLH202 (AJ486857)	99%

*ars*	*trxB*, *arsH*, *B*, *C*, *R*, *C*	pALWED2.1 pALWED3.1	*A. johnsonii* XBB1 (CP010350)	78%

*cop*	*copD*, *C*, *F1*, *S*, *R*, *A*, *B*	pALWED2.1 pALWED3.1 pALWVS1.1 pALWEK1.1	*A. baumannii* AB0057 (CP001182)	76%

*czc1*	*czcC*, *B*, *A*, *D*	pALWVS1.1 pALWEK1.1	*A. guillouiae* NBRC 110550 (AP014630)	74%

*czc2*	*czcC*, *B*, *A*, *D*, *rcnR*, *nreB*	pALWED1.1	*A. johnsonii* XBB1 (CP010350)	82%

*czc3*	*czcC*, *B*, *A*, *D*	pALWED3.1	*Moraxella osloensis* CCUG 350 (CP014234)	97%

*chr1*	*chrA-chrB*	pALWED1.3 pALWED3.5 pALWEK1.5	*A*. sp M131, pM131-6 (JX101643)	99%

*chr2*	*chrA-chrB*	pALWED1.1	*A*. sp M131, pM131-6 (JX101643)	71%

^*∗*^ *merB* gene is present only in the plasmid pALWED1.1.

**Table 4:** Heavy metal resistance determinants in chromosomes and plasmids in strains of *A. lwoffii*.

Strain, AC	Localization of genes	Presence of resistance genes and size of corresponding protein (aa)									
		ChrA	ChrB	CzcA	CzcB	CzcC	CzcD	CopA	CopB	CopC	CopD
NCTC 5866 = CIP64.10 = NIPH 512, APQS00000000	Chromosome	401	—	—	408	441	305 + 313	588	319	—	—
	Plasmid^*∗*^	449	308	1054	—	—	—	580	329	126	308

NIPH 478, APQU00000000	Chromosome	401	—	—	—	—	313	588	314	—	—
	Plasmid^*∗*^	449	308	1052	411	455	299	nd	306	126	308

NIPH 715, APOT00000000	Chromosome	401	—	—	—	—	313	560	315	—	—
	Plasmid^*∗*^	—	—	1052	416	455	284	603	339	126	308

ATCC 9957 = CIP 70.31, APQT00000000	Chromosome	401	—	—	—	—	313	588	314	—	—
	Plasmid^*∗*^	449	308	1052	411	455	299	nd	306	126	308

TG19636, AMJG00000000	Chromosome	401	—				259	588	339	—	—
	Plasmid^*∗*^	—	—	1052	416	455	305	617	306	126	308

ЕД23-35	Chromosome	401	401	—	—	—	259	580	339	—	—
	Plasmid	449	308	1042	491	399	315	—	—	—	—

VS15	Chromosome	401	—					585	315	—	—
	Plasmid	—	—	1052				637	306	126	308

EK30A	Chromosome	401	—	—	—	—	259	585	315	—	—
	Plasmid	449	308	1052	436	455	299	637	306	126	308

^*∗*^Contig identified as a plasmid with a high probability; nd: not determined.
