# Supplementary material for: Resistance of Permafrost and Modern Acinetobacter lwoffii Strains to Heavy Metals and Arsenic Revealed by Genome Analysis
Source: Biomed Res Int. 2016 Oct 4;2016:3970831. doi: 10.1155/2016/3970831 (PMC5067307; doi:10.1155/2016/3970831)
Supplement: Supplementary file 1 — The Supplementary Material contains 4 tables. The data presented in Table S1 indicate that determinants revealed on the plasmids of modern A.lwoffii clinical strains are closely related to those of the ancient A. lwoffii plasmids. Table S2 contains the data on the structure and distribution of chromosomal ars operons in A. lwoffii strains. Tables S3 and S4 contain the information on comparative genome size of the strains of A.lwoffii and A. baumannii, respectively. [file 3970831.f1.pdf]

Table S1. Contigs of clinical *A.lwoffii* strains carrying heavy metal resistance determinants closely related to determinants of ancient plasmids

Strain of *A. lwoffii*	Contigs with determinants of resistance to				
	mercury	arsenicum	chromium	copper	cobalt/zinc/cadmium
NCTC 5866	**adgTz-supercont2.5.C9 14351 bp, AYHO01000009**	**adgTz-supercont2.5.C9, 14351 bp, AYHO01000009**	**adgTz-supercont2.5.C9, 14351 bp, AYHO0100000]**	adgTz-supercont2.6.C10, 14188 bp, AYHO01000010	-
NIPH 478	-	**acLZS-supercont1.3.C4 131122 bp*, APQU01000004**	acLZS-supercont1.9.C26, 20292 bp, APQU01000026	acLZS-supercont1.3.C3, 17981 bp, APQU01000003	**acLZS-supercont1.3.C4 131122 bp, APQU01000004**
NIPH715	**acLrE-supercont1.22.C69 , 33082 bp, APOT01000069**	**acLrE-supercont1.22.C69, 33082 bp, APOT01000069**	-	**acLrE-supercont1.22.C69, 33082 bp, APOT01000069**	acLrE-supercont1.22.C68, 79564 bp, APOT01000068
CIP 70.31	-	-	acLsr-supercont1.3.C10, 7846 bp, APQT01000010	**acLsr-supercont1.11.C59 64431 bp * APQT01000059**	**acLsr-supercont1.11.C59, 64431 bp APQT01000059**
TG19636	-	-	-	-	TG19636 223, 13100 bp, AMJG01000222
SH145	-	-	-	-	cont1.149, 25730 bp, ACPN01000149

NBRC 109760	**ALW03_CON00057, 14170 bp, BBSQ01000057**	**ALW03_CON00057 14170 bp, BBSQ01000057**	**ALW03_CON00057, 14170 bp, BBSQ01000057**	ALW03_CON00054, 15382 bp, BBSQ01000054; ALW03_CON00074, 3744bp*, BBSQ01000074	**-**

*not complete

Table S2. Genes of *ars* operon (*trxB*-*arsH*- *arsB*-*arsC*-*arsR*-*arsC*) revealed on different contigs (presumably chromosome regions) of *A. lwoffii* clinical strains

Strain	Contig [AC]	Genes	Identity (%) *
A. lwoffii NCTC 5866	0087 [AIEL01000087]	*arsH-arsB-arsC*	77%; 68%; 74%
	0016 [AIEL01000016]	*arsC-arsR*	83%; 68%
	0095 [AIEL01000095]	*trxB*	77%
A. lwoffii NIPH 478	aclZS-supercont1.5.C10 [APQU01000010]	*arsC-arsR*	83%; 68%
	aclZS-supercont1.5.C12 [APQU01000012]	*trxB*	77%
A. lwoffii NBRC 109760	ALW03_CON00003 [BBSQ01000003]	*arsC-arsR*	83%; 68%
	ALW03_CON00040 [BBSQ01000040]	*arsH-ars-arsC*	76%; 78%; 68%
	ALW03_CON00021 [BBSQ01000021]	*trxB*	77%
A. lwoffii ED23-35	7	*arsH-arsB-arsC-arsR-arsC*	86%; 87%, 81%, 75%, 68%
	36	*trxB*	<40%**
A. lwoffii VS15	18	*arsH-arsB-arsC-arsR-arsC*	86%; 87%, 81%, 75%, 67%
	27	*arsH*	92%
	11	*trxB*	<40%**

*in relation to nucleotide sequences (100%) of ars genes from pALWED2.1

** identity at the amino acid sequence level

Table S3. Genome size of *A. lwoffii* clinical isolates

Strain	AC	Whole genome size
NCTC 5866 (= CIP64.10 = NIPH 512	NZ_AIEL0100000000.1	3.353.128
NIPH 512	NZ_AYHO00000000.1	3,382,003
NIPH 478	NZ_APQU00000000.1	3,276,129
NIPH 715	NZ_APOT00000000.1	3,400,693
CIP 70.31	NZ_APQT00000000.1	3,546,109
TG19636	NZ_AMJG00000000.1	3,497,784
SH145	NZ_ACPN00000000.1	3,347,001
WJ10621*	NZ_CM001194.1	3,419,011<br>3,184,320**
Average size of whole genome		3,400,407

*Carries a plasmid pNDM-BJ01 (47,274 bp)[NC_019268.1] with the gene bla $_{NDM-1}$;
**Main genome size

Table S4. Characteristics of genomes of *A. baumannii* strains

Strain	AC	Main genome size	No of plasmids	Plasmid size, (bp)	Whole genome size, (bp)
AB0057	CP001182	4,050,513	1	8,729	4,059,242
Ab04-mff	CP012006	3,935,688	2	169,023; 87,569	4,192,280
AB307-0294,	CP001172	3,760,981	-	-	3,760,981
AB5075-UW	CP008706	3,972,672	3	83,610; 8,731; 1,967	4,066,980
AC29	CP007535	3,851,648	2	74,749; 8,737	3,935,134
ACICU	CP000863	3,904,116	2	64,366; 28,279	3,996,761
ATCC 17978	CP000521	3,976,747	2	13,408; 11,302	4,001,457
ATCC 17978-mff	CP012004	3,857,743	1	148,955	4,006,698
AYE	CU459141	3,936,291	4	94,413; 9,661; 5,644; 2,726	4,048,735
BJAB07104	CP003846	3,951,920	2	70,170; 20,139	4,042,229

BJAB0715	CP003847	4,001,621	1	52,268	4,053,889
BJAB0868	CP003849	3,906,795	3	70,167; 20,139; 8,721	4,005,822
CIP70.10	LN865143	3,928,513	1	7,742	3,936,255
D36	CP012952	4,063,596	4	47,457; 9,276; 6,078; 4,754	4,131,161
D1279779	CP003967	3,704,284	1	7,416	3,711,700
IOMTU433	AP014649	4,000,970	1	189,354	4,190,324
KBN10P02143	CP013924	4,086,879	1	52,517	4,139,396
MDR-TJ	CP003500	3,964,912	2	110,967; 77,528	4,153,407
MDR-ZJ06	CP001937	3,991,133	1	20,301	4,011,434
NCGM 237	AP013357	4,021,920	-	-	4,021,920
PKAB07	CP006963	4,233,806	1	8,805	4,242,611
R2090	LN868200	3,819,158	-	-	3,819,158
R2091	LN997846	3,939,746	1	7,742	3,947,488
SDF	CU468230	3,421,954	3	25,014; 24,922; 6,106	3,477,996

TCDC-AB0715	CP002522	4,138,388	2	70,894; 8,731	4,218,013
TYTH-1	CP003856	3,957,368	1	65,890	4,023,258
XH386	CP010779	4,087,157	1	112,157	4,199,314
YU-R612	CP014217	4,075,545	2	74,241; 5,465	4,155,251
ZW85-1	CP006768	3,763,012	2	113,866; 48,368	3,925,246
1656-2	CP001921	3,940,614	2	74,451; 8,041	4,023,106
6200	CP010397	3,902,527	3	114,848; 47,274; 9,327	4,073,976
average value	-	3,939,942	2	-	4,018,426
